# Selection of Potential Therapeutic Bacteriophages that Lyse a CTX-M-15 Extended Spectrum β-Lactamase Producing *Salmonella* *enterica* Serovar Typhi Strain from the Democratic Republic of the Congo

**DOI:** 10.3390/v10040172

**Published:** 2018-04-03

**Authors:** Elene Kakabadze, Khatuna Makalatia, Nino Grdzelishvili, Nata Bakuradze, Marina Goderdzishvili, Ia Kusradze, Marie-France Phoba, Octavie Lunguya, Cédric Lood, Rob Lavigne, Jan Jacobs, Stijn Deborggraeve, Tessa De Block, Sandra Van Puyvelde, David Lee, Aidan Coffey, Anahit Sedrakyan, Patrick Soentjens, Daniel De Vos, Jean-Paul Pirnay, Nina Chanishvili

**Affiliations:** 1 Eliava Institute of Bacteriophage, Microbiology & Virology and Tbilisi State University, Gotua Street 3, Tbilisi 0160, Georgia; elene.kakabadze@pha.ge (E.K.); khatuna.makalatia@pha.ge (K.M.); nino.grdzelishvili.3@iliauni.edu.ge (N.G.); nata.bakuradze@pha.ge (N.B.); mgoderdzishvili@pha.ge (M.G.); iakusradze@pha.ge (I.K.); 2 National Institute for Biomedical Research, University Teaching Hospital of Kinshasa, Kinshasa, Democratic Republic of the Congo; mfphoba@hotmail.com (M.-F.P.); octmetila@yahoo.fr (O.L.); 3 Department of Microbiology, University Teaching Hospital of Kinshasa, Kinshasa, Democratic Republic of the Congo; 4 KU Leuven, Laboratory for Gene Technology, B-3000 Leuven, Belgium; cedric.lood@kuleuven.be (C.L.); rob.lavigne@kuleuven.be (R.L.); 5 Centre of Microbial and Plant Genetics, KU Leuven, B-3000 Leuven, Belgium; 6 Department of Clinical Sciences, Institute of Tropical Medicine, B-2000 Antwerpen, Belgium; jjacobs@itg.be (J.J.); Patrick.Soentjens@mil.be (P.S.); 7 Department of Microbiology and Immunology, KU Leuven, B-3000 Leuven, Belgium; 8 Department of Biomedical Sciences, Institute of Tropical Medicine, B-2000 Antwerpen, Belgium; sdeborggraeve@itg.be (S.D.); tdeblock@itg.be (T.D.B.); svanpuyvelde@itg.be (S.V.P.); 9 Wellcome Trust Sanger Institute, Hinxton, Cambridge CB10 1SA, UK; 10 Department of Biological Sciences, Cork Institute of Technology, Cork T12 P928, Ireland; david.lee@mycit.ie (D.L.); aidan.coffey@cit.ie (A.C.); 11 Institute of Molecular Biology, 0014 Yerevan, Armenia; sedanahit@gmail.com; 12 Center for Infectious Diseases ID4C, Queen Astrid Military Hospital, B-1120 Brussels, Belgium; 13 Laboratory for Molecular and Cellular Technology, Queen Astrid Military Hospital, B-1120 Brussels, Belgium; DanielMarie.DeVos@mil.be (D.D.V.); jean-paul.pirnay@mil.be (J.-P.P.)

**Keywords:** typhoid fever, *Salmonella* Typhi, extended-spectrum beta lactamases (ESBL), Democratic Republic of the Congo, bacteriophages

## Abstract

Recently, a *Salmonella* Typhi isolate producing CTX-M-15 extended spectrum β-lactamase (ESBL) and with decreased ciprofloxacin susceptibility was isolated in the Democratic Republic of the Congo. We have selected bacteriophages that show strong lytic activity against this isolate and have potential for phage-based treatment of *S*. Typhi, and *Salmonella* in general.

Although effective oral antibiotics used to be readily available, today antimicrobial resistance in *Salmonella* Typhi is becoming an increasingly serious public health concern, especially in low- and middle-income countries (LMICs). Resistance to all first-line antimicrobials used in the treatment of *S.* Typhi infections emerged sequentially, leading to multidrug resistance (MDR) in the 1990s, and more recently, high levels of fluoroquinolone resistance in South Asia [1]. Recent data from the Typhoid Fever Surveillance in Africa Program (TSAP) indicates that the incidence rate for typhoid fever in Africa has been underestimated and is equal to, or even greater than, incidences reported in Asia [2]. Therefore, extended spectrum β-lactamase (ESBL) producing *Enterobacteriaceae* and fluoroquinolone resistant *Salmonellae* were included in the high-priority tier of the recent WHO priority list of antibiotic-resistant bacteria [3].

In general, it is acknowledged that global antimicrobial resistance (AMR) poses a fundamental long-term threat to human health, the production of food, and sustainable development. Based on scenarios of rising drug resistance for only six pathogens, experts estimated that by 2050, up to 10 million people could die every year from the effects of AMR and it could also impose an economic burden of US$100 trillion [4]. Recently, the UN committed to supporting the development of new antimicrobial agents and therapies [5].

Phage therapy is one of the promising “new” treatments that has been increasingly featured [6]. Bacteriophages (phages in short) are naturally occurring viruses of bacteria. Since the early phases of evolution, phages have controlled bacteria on our planet. In the early twentieth century, humans discovered them and immediately applied them to medicine. This was especially true in the former Soviet Union, where the use of phages continued after the advent of commercial antibiotics [7]. They can be selected to kill only certain bacteria of concern (e.g., bacteria causing infectious diseases) while leaving non-pathogenic bacteria and mammalian cells unharmed. As such, they can be effective against antibiotic-resistant bacteria and, in contrast to broad-spectrum antibiotics, spare the gut microbiota, which could particularly benefit malnourished and immunocompromised patients. In addition, phages can be easily isolated from environmental sources such as river or sewage water, using basic tools available in LMICs [8].

In 2017, a case of typhoid fever in a six-year-old boy in the Democratic Republic of the Congo (DRC) caused by an *S*. Typhi isolate producing CTX-M-15 extended spectrum β-lactamase (ESBL) and showing decreased ciprofloxacin susceptibility, was reported [9]. CTX-M-15 is part of the M1 group that includes six plasmid-mediated enzymes [10]. This isolate, named Typhi 10040_15, was sent to the Eliava Institute of Bacteriophage, Microbiology & Virology (EIBMV) in Tbilisi (Georgia) to determine phage susceptibility. The 14 *Salmonella* phage clones of the Eliava R&D collection and five batches of the commercial phage cocktail “INTESTI phage” were tested. Phage screening against this *S.* Typhi strain was performed at the EIBMV’s BSL-2 Plus laboratory, meeting the required safety requirements. The fourteen phage clones were isolated from the river Mtkvari in Tbilisi, from the Black Sea (Batumi) and from the Tbilisi sewage water supply system in the period 2013–2017 (Table 1). The five tested INTESTI phage batches were #M 067 (produced in July 2017), #M2 901 (November 2017), #84 of (February 2017), #82 (January 2017), and #78 (December 2016).

As in many other pathogenic bacteria, temperate phages contribute to virulence in *Salmonella enterica* through the acquisition and exchange of virulence factors [11]. To assert the strictly lytic nature of these phages, high-resolution genome maps of 12 of the 14 individual phages were obtained using nanopore sequencing [12]. A pooled library consisting of barcoded genomic DNA of the phages was prepared using native barcodes and the 1D ligation kit from Oxford Nanopore Technology (ONT). The result was then sequenced on a MinION device, equipped with an R9.4 flowcell. For the data analysis, Albacore v2.1 (ONT, Oxford, UK) was used for base-calling the reads, followed by porechop v0.2.1 (https://github.com/rrwick/Porechop) in order to remove barcode sequences. Genome map assembly was performed with Canu v1.6 (https://github.com/marbl/canu) [13]. All the assembled genomes were subsequently processed with Racon v0.5 for better consensus sequences [14], and nanopolish v0.8.3 (https://github.com/jts/nanopolish) for higher accuracy of base-called nucleotides in the sequences. Considering the intrinsic properties of nanopore sequencing, together with the run coverage (30× to 60×), we define these assemblies as high-resolution phage maps, rather than fully accurate genome sequences. Known homologous phage isolates were first located using the blastn tool on the NCBI nucleotide database [15]. For each of our new isolates, the closest match (highlighted in bold) in terms of query coverage and identity was identified and the corresponding genome downloaded. The genomic distance between all the pairs of phages was calculated using Mash [16] and the resulting distance matrix was used to build the clustering tree (Figure 1) with the hclust function found in the R stats package [17]. No known toxin genes were present and the genomes did not contain recognizable integrase genes, corroborating the lytic nature of these bacteriophages. Sequences were submitted to GenBank (Accession numbers: MG969404-15).

Morphological analysis using Transmission Electron Microscopy (TEM) of the phage clones confirmed their general classification (Table 1). Purification and staining of the samples was performed according to Hans-W. Ackermann [18] and preparations were examined with JEOL-JEM-1400 TEM (Figure 2). Phages belonged to the families of the *Siphoviridae* (*n* = 6), *Myoviridae* (*n* = 6) and *Podoviridae* (*n* = 2). The genome map-based grouping allowed us to further assign these phage clones to individual phage species/genera, as indicated in Table 1.

The host range of the phages was assessed by screening their lytic activity and using the spot test against 118 clinical and 121 veterinary *Salmonella*. spp. isolates from Georgia (20), Armenia (71), Germany (7), and Ireland (141). These isolates belonged to the following serotypes: *S*. Typhimurium (95), *S*. Enteritidis (45), *S*. Dublin (23), *S*. Anatum (11), *S*. Infantis (9), *S*. Newport (8), *S*. Derbey (8), *S*. Bredney (5), *S*. Branderburg (3), *S*. Germinara (2), *S*. Uganda (2), *S*. Senftenberg (2), *S*. Kentucky (2), *S*. Reading (2), *S*. Parat. B (2), *S*. Java (1), *S*. Bareilly (1), *S*. Virchow (1), *S*. Goldcost (1), *S*. Kottbus (1), *S*. Agona (1), and *S*. Poona (1). Thirteen isolates were not attributed to any known serotypes. Two hundred microlitres of *S. enterica* mid-log phase cultures were mixed with 5 mL of lukewarm 0.6% Lysogeny Broth (LB, Merck, Darmstadt, Germany) agar and overlaid on LB agar plates. The LB broth consisted of 10 g peptone from casein, 5 g yeast extract and 10 g NaCl in 1 L of deionized water. After the plates had cooled, 5 µL of the phage clones, with a titer of 10^7^ plaque forming units (pfu)/mL was spotted on the lawn. Drops were air-dried and plates were incubated for 18 h at 37 °C. After the incubation, the plates were checked for zones of clearance resulting from phage activity [19]. The host range of the phages varied from 12 to 81% of the *S. enterica* strains (Table 1). It should be noted that the spot test is usually performed to determine bacterial susceptibility and host range using as many bacterial strains as possible because this method is simple, quick and inexpensive. A significant part of bacterial cell killing can be due to “lysis from without”, i.e., the destruction of bacterial cells by the adherence of a sufficiently high number of phages to the bacterial cell, and the destruction of an essential cell wall structure by an extracellular lytic enzyme with subsequent lysis, but without phage replication.

The lytic activity of the 14 phage clones and of the five INTESTI phage batches against the Typhi 10040_15 isolate from the DRC was evaluated using the spot test and the streak method. For the streak method, mid-log phase *S*. Typhi was plated as a single line on an agar plate, air dried and 5 µL of single phage clones (titer 10^7^ pfu/mL) was dropped on the bacterial line. Drops were air-dried and plates were incubated for 18 h at 37 °C. After incubation, plates were checked for zones of clearance resulting from phage activity [20]. Twelve out of 14 phage clones and three out of five batches of the commercial preparation INTESTI phage formed clear lysis zones on the CTX-M-15, producing an *S.* Typhi strain from the DRC. This occurred in the streak method, as well as in the spot test. The batches #M 067 and #M2 901 showed the strongest lytic activity with clear confluent zones, while batch #84 only developed weakly distinguishable discrete lytic zones. Only two phage clones, phage GE_vB_MG (*Myoviridae*, *Vequintavirinae*, *Se1virus*, *Salmonella virus SE1*) and phage GE_vB_TR, a potential lysogenic phage (*Podoviridae*, *P22virus*), showed no activity against the Typhi 10040_15 isolate (Table 1).

To assess the ability of phages to multiply inside the bacterial cells (creating phage plaques), the activity of the 14 phage clones (but not the INTESTI phage batches) was tested using the method of Gratia. A 200 µL mid-log phase culture of *S*. Typhi and 1 mL of a ten-fold diluted phage suspension, ranging from 10^6^ to 10^10^ pfu/mL, were mixed with 5 mL of lukewarm 0.6% LB agar and overlaid on LB agar. Plates were incubated at 37 °C for 18 h and checked for plaque formation [21]. All phage clones, with the exception of three (GE_vB_N8, GE_vB_HIL and GE_vB_M1), were found to form plaques on the bacterial lawn (Table 1).

Finally, to confirm the ability of the phages to lyse the DRC *S*. Typhi strain in aqueous solutions, the lytic activity of the 14 phage clones was determined using Appelman’s method. The 14 phage clones were diluted ten-fold to range from 10^6^ to 10^10^ pfu/mL in 5 mL of LB broth and inoculated with 150 µL (10^9^ cfu/mL) overnight *S*. Typhi culture. Mixtures were incubated at 37 °C without shaking and the turbidity of the samples was checked visually after 6, 18 and 24 h. As reference and control samples, phage-free bacterial culture and diluted phages without bacteria were tested under the same conditions [22]. Ten phage clones showed activity at different time points and concentrations, five of which showed the ability to lyse the CTX-M-15 producing *S*. Typhi isolate without forming phage-resistant mutants after 24 h of incubation, which is indicative of their inherent ability to limit the growth of phage-resistant mutants during phage therapy: GE_vB_N3, GE_vB_N5, GE_vB_N8, GE_vB_NS7 and GE_vB_HIL (Table 1). It should be noted that phages GE_vB_MG and GE_vB_TR are not active according to the spot and the streak tests, while still forming plaques according to the method of Gratia. The difference between these two methods is not that uncommon and could, for example, be caused by differences between phages’ “lysis from without” (accentuated in the spot test) and phage infection, or “lysis from within” (accentuated in the Gratia test) capabilities. In other words, some phages could be less efficient in adhering to, and entering bacterial cells, while being very efficient once the normal lytic cycle is initiated. The spot test and the streak method are rapid ways to check whether a phage can infect a bacterium by trickling small droplets of the phage suspensions to be tested on a plate prepared with a bacterial isolate. Limitations of these tests compared with the Gratia and Appelmans methods are that clear zones on the bacterial lawn (a positive test), may be the result of abortive infection or lysis from without, both forming clear zones without new phages being produced.

The evolution of bacterial resistance to phages is often observed in vitro. Phages have evolved multiple strategies to overcome the antiviral mechanisms they encounter when infecting bacterial cells [23]. In experimental settings, phage-resistant bacteria emerged rapidly, but often at significant fitness costs, shown by the reduced growth rate in the absence of phages [24]. The in vivo evolution of bacterial resistance to phages in human clinical practice, however, was poorly documented until now. It has been suggested that phages could be combined with antibiotics to improve phage activity (synergy) [25].

In conclusion, in a short time frame (two days), at least five phage clones from the Eliava collection were found to exhibit excellent in vitro lytic activity against the ESBL producing *S*. Typhi isolate from the DRC. Phages can be considered a potential additional tool for the treatment of MDR *Salmonella* infections and a (food) decontamination agent. Antimicrobials that address foodborne diseases are particularly important for LMICs as many of them lack reliable cold chain infrastructure and adequate hygiene practices [8]. In Western countries, several phage products are currently approved for the control of food pathogens, including *Salmonella*. In addition, phage preparations can be developed and produced faster and cheaper than conventional drugs. They can also be (freeze-)dried [26] so that they require no refrigeration [8].

## Figures and Tables

**Figure 1:**
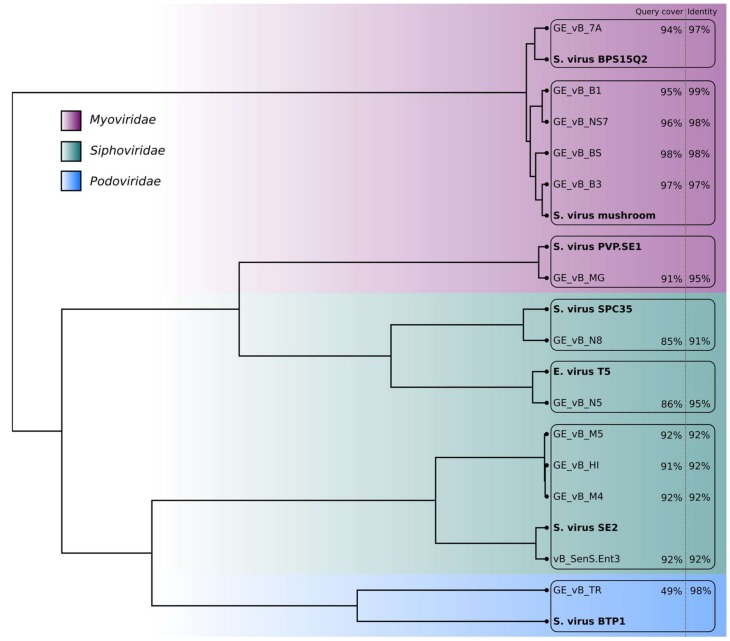
Clustering tree based on the genomic distance matrix generated for the *Salmonella* phages from the Eliava R&D collection and their closest matches (in bold) in the NCBI database. No genome maps were obtained for phages GE_vB_N3 (Siphovirus) and GE_vB_M1 (Podovirus).

**Figure 2:**
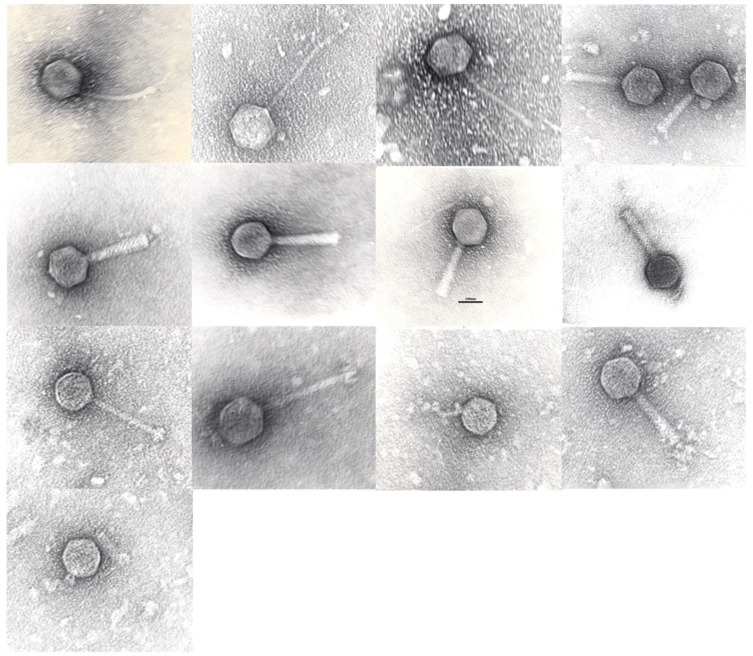
Tansmission electron micrographs of phages in left->right, top->bottom order: GE_vB_N3, GE_vB_N5, GE_vB_N8, GE_vB_MG, GE_vB_BS, GE_vB_B1, GE_vB_B3, GE_vB_NS7, GE_vB_M4, GE_vB_M5, GE_vB_TR, GE_vB_7A, GE_vB_M1. Scale bar, 100 nm.

**Table 1:** Characteristics of 14 *Salmonella* phages from the Eliava collection tested on the Typhi 10040_15_DRC_2015 isolate from the Democratic Republic of the Congo (DRC).

Nr	Name	GenBank Accession Numbers	Source	Isolation Year	Host Strain	Family	Homology to Other Phages	Host Range (%) *All Serovars/*S*. Enteritidis/*S*. Typhimurium/*S*. Dublin (Total Number of Tested Isolates)	Lytic Activity on the Typhi 10040_15_DRC_2015 Isolate							
									Streak Method	Spot Test	Gratia’s Method	Appelmans’ Method				
												Titer (pfu/mL)	Dilution	6 h	18 h	24 h
001	GE_vB_N3	ND	Mtkvari river water	2013	*S.* Enteritidis 3	Siphoviridae	ND	78/98/73/87 (239)	+	+	+	10^9^	−1	−	−	+
												10^8^	−2	−	−	+
												10^7^	−3	−	+	+
002	GE_vB_N5	MG969412	Mtkvari river water	2013	*S.* Enteritidis 3	Siphoviridae	*E. coli T5 strain ATCC 11303-B5*	45/80/25/74 (239)	+	+	+	10^8^	−1	−	+	+
												10^7^	−2	−	+	+
												10^6^	−3	−	+	+
003	GE_vB_N8	MG969413	Mtkvari river water	2013	*S.* Enteritidis 3	Siphoviridae	*phage SPC35*	65/84/67/78 (239)	+	+	−	10^9^	−1	−	−	+
												10^8^	−2	−	−	+
												10^7^	−3	−	+	+
004	GE_vB_MG	MG969411	Tbilisi sewage water	2013	*S.* Enteritidis 3	Myoviridae	*S. phage PVP-SE1*	47/49/59/43 (239)	−	−	+	10^9^	−1	−	−	−
												10^8^	−2	−	−	−
												10^7^	−3	−	−	−
005	GE_vB_BS	MG969407	Black Sea water	2013	*S.* Typhimurium 4	Myoviridae	*S SPT-1, partial genome*	81/96/93/83 (239)	+	+	+	10^10^	−1	+	−	−
												10^9^	−2	+	−	−
												10^8^	−3	+	−	−
006	GE_vB_B1	MG969405	Mtkvari river water	2013	*S.* Typhimurium 6	Myoviridae	*S. phage Mushroom*	80/93/83/87 (239)	+	+	+	10^9^	−1	+	−	−
												10^8^	−2	+	−	−
												10^7^	−3	+	−	−
007	GE_vB_B3	MG969406	Mtkvari river water	2013	*S.* Typhimurium 6	Myoviridae	*S. phage Mushroom*	81/98/73/87 (239)	+	+	+	10^9^	−1	+	−	−
												10^8^	−2	+	−	−
												10^7^	−3	+	−	−
008	GE_vB_NS7	MG969414	Raw cow milk	2015	*S.* Typhimurium 6	Myoviridae	*S. phage Mushroom*	75/91/81/78 (239)	+	+	+	10^9^	−1	+	−	−
												10^8^	−2	−	+	−
												10^7^	−3	−	+	+
009	GE_vB_M4	MG969409	Black Sea water	2016	*S.* Enteritidis 232	Siphoviridae	*S. phage vB_SenS-Ent3*	23/64/18/22 (218)	+	+	+	10^10^	−1	+	−	−
												10^9^	−2	+	−	−
												10^8^	−3	−	−	−
010	GE_vB_M5	MG969410	Black Sea water	2016	*S.* Enteritidis 407	Siphoviridae	*S. phage vB_SenS-Ent3*	33/66/26/61 (218)	+	+	+	10^8^	−1	+	−	−
												10^7^	−2	+	−	−
												10^6^	−3	+	−	−
011	GE_vB_TR	MG969415	Mtkvari river water	2017	*S.* Typhimurium 641	Podoviridae	*S. phage BTP1*	40/90/28/59 (141)	−	−	+	10^9^	−1	−	−	−
												10^8^	−2	−	−	−
												10^7^	−3	−	−	−
012	GE_vB_HIL	MG969408	Mtkvari river water	2017	*S.* Enteritidis 765	Siphoviridae	*S. phage vB_SenS-Ent3*	58/81/75/77 (141)	+	+	−	10^10^	−1	+	−	−
												10^9^	−2	−	−	−
												10^8^	−3	−	+	+
013	GE_vB_7A	MG969404	Mtkvari river water	2017	*S.* Typhimurium 1328	Myoviridae	*S. phage BPS15Q2*	37/62/28/23 (141)	+	+	+	10^8^	−1	−	−	−
												10^7^	−2	−	−	−
												10^6^	−3	−	−	−
014	GE_vB_M1	ND	Black Sea water	2016	*S.* Enteritidis 104	Podoviridae	ND	12/20/19/0 (77)	+	+	−	10^9^	−1	−	−	−
												10^8^	−2	−	−	−
												10^7^	−3	−	−	−

* The host range of the phages was determined for the total *Salmonella* strain collection (all serovars), with the total number of strains indicated between brackets, and for the three main serovars (*S*. Enteritidis/*S*. Typhimurium/*S*. Dublin) separately. ND, not done; “+”, phage lytic activity; “−”, no phage lytic activity; *S. phage*: *Salmonella phage*.
